# Field testing project to pilot World Health Organization global eye health indicators in Latin America: lessons learned thus far

**Published:** 2016

**Authors:** Kristen A Eckert, Marissa J Carter, Van C Lansingh, Juan C Silva, Joan McLeod-Omawale

**Affiliations:** Independent Consultant: Strategic Solutions and International Agency for the Prevention of Blindness, Tapachula, Mexico.; President: Strategic Solutions, Cody, USA. **mcarter@strategic-solutions-inc.com**; Medical Officer Latin America: HelpMeSee, New York, USA.; Regional Adviser for Visual Health: Pan-American Health Organization/World Health Organization, Bogota, Colombia; Latin American and Caribbean Regional Program Director: Orbis International, New York, USA.

## Global eye health indicators

The World Health Organisation (WHO) global action plan ‘Universal eye health: a global action plan 2014–2019’ (the GAP)^1^ includes a global target to reduce the prevalence of avoidable visual impairment by 25% by 2019 (compared to the 2010 baseline). Progress against this target will be measured by three core indicators, which the WHO suggest should be reported on regularly by member states:

- Prevalence and causes of visual impairment
- Cataract surgical services: cataract surgical rate (CSR) and cataract surgical coverage
- Number of eye health professionals by cadre (ophthalmologists, optometrists and allied eye health personnel).

A project is underway in Latin America to strengthen the collection and reporting of eye health indicators in collaboration with ministries of health, national VISION 2020 or prevention of blindness committees, and national professional societies in five countries: Chile, Honduras, Mexico, Peru, and Uruguay. Secondary objectives include evaluating the feasibility of accurate data collection, the reliability of the indicators, and the adequacy of the metrics used to define the indicators, as well as barriers to obtaining these data.

The project has four phases: 1. Situational analysis, 2. Implementation, 3. Data collection and 4. Final evaluation.

During phase 1, public and private sector representatives from each country completed a situational analysis of data collection and reporting in their respective countries. Data were presented during the First Latin American Global Indicators Workshop in Lima, Peru, in March, 2014. In phase 2, country teams developed and implemented improved data collection strategies and global indicator work plans (March 2014-January 2015).

For phase 3 (ongoing), teams should use the tools and lessons learned from phase 1 and 2 to collect and report 2015 eye health data. The final evaluation (phase 4) will take place in 2016 and will conclude with development of a research protocol to implement the global indicators in other regions of the world.

What follows are the lessons learned thus far.

### 1. Inter-sectoral and inter-institutional cooperation is essential to the success of complete and accurate data collection. Without a functioning national VISION 2020 or prevention of blindness committee, objectives are much harder to achieve.

The active participation of all potential stakeholders (groups and individuals with an interest, or who are involved, in data collection) is necessary. The national committees are the appropriate channel for this process. Previously, the committees in each country varied in function, with some committees meeting on a regular basis to advance eye health planning and implementation, while others were either dormant in recent years or only active when commissioned to perform a project, such as an epidemiological study. An important outcome of this project has been that national committees were reorganised in 2014 to incorporate global indicator data collection among their ongoing functions.

### 2. Both a complete, national data registry and a data validation process are needed for successful outcomes.

The gold standard of CSR data collection is when every cataract operation is reported to the ministry of health. Although ministries of health may have good data from the public sector, the estimation of number of operations conducted in the private sector is poor. The most effective solution to this problem is legislation that mandates the reporting of all cataract operations, with cross-referencing from intra-ocular lens (IOL) sales and importation data.

This requires further inter-institutional and inter-sectoral cooperation. Argentina has spearheaded this effort, with new legislation in 2014 that mandates the reporting of all cataract operations to their ministry of health (Resolution No. 8/14) and requires that the sales, distribution, and surgical implantation of all IOLs in the country are registered on a government website for medical goods and technology.^2^

### 3. The lack of standardised indicators for different eye health personnel roles is a barrier to data collection.

Across the globe, there is no standardised, defined eye care team, nor is it defined by the WHO GAP. Most countries will collect different human resources data. Each ministry of health defines their country's official, legally recognised health care roles in their policy. Optometry is not recognised in some countries and allied eye health personnel vary. For example, although Chile does not allow optometry, there are trained medical technologists, working in ophthalmology, who can provide refractive services. There is therefore a need for minimum competency requirements and regional certification for human resources in eye care.

## Moving forward

Strong, clear coordination and continuous knowledge sharing between all project stakeholders are essential if we are to learn from each country's experience and ensure a successful final evaluation of global indicators.

## Acknowledgements

The project is sponsored by the Fred Hollows Foundation, with additional financial support from Orbis International and the International Agency for the Prevention of Blindness (IAPB). The project is managed by Strategic Solutions, Inc, with the collaboration of the Pan American Health Organization (PAHO), Orbis International, and IAPB.
